# Exogenous treatment with N-acetylglutamic acid confers tolerance to heat stress in plants

**DOI:** 10.5511/plantbiotechnology.23.1211a

**Published:** 2024-03-25

**Authors:** Takeshi Hirakawa, Seia Tanno, Kazuaki Ohara

**Affiliations:** 1 Kirin Central Research Institute, Kirin Holdings Company, Ltd., 26-1, Muraoka-Higashi 2, Fujisawa, Kanagawa 251-8555, Japan

**Keywords:** chemical priming, epigenetic regulation, heat stress, histone acetylation, N-acetylglutamic acid

## Abstract

Heat stress, which occurs when temperatures exceed the optimal range for growth, challenges the maintenance of crop yield because it disrupts plant homeostasis at the cellular and developmental levels. Chemical priming, which can activate the response to environmental stress using chemical compounds, is a promising method of maintaining plant growth under stressful conditions. Recently, we found that the non-proteogenic amino acid N-acetylglutamic acid (NAG) confers tolerance to oxidative stress through the activation of genes related to scavenging reactive oxygen species in plants. However, it has been unknown whether NAG alleviates environmental stress except oxidative stress. Here, we revealed that the response to heat stress was enhanced by exogenous treatment with NAG in plants. NAG alleviated the reduction in chlorophyll content induced by heat stress in *Arabidopsis thaliana*. Gene expression analysis showed that NAG activates the transcription factor *HSFA2*, which is regarded as a master regulator of the transcriptional cascade in response to heat stress. NAG induces histone H4 acetylation, an active histone modification, at the *HSFA2* locus, suggesting that NAG could activate the expression of *HSFA2* based on epigenetic modifications such as histone acetylation. Additionally, we found that *Oryza sativa* treated with NAG showed tolerance to heat stress. These results suggest that NAG could be used for chemical priming in the maintenance of plant growth under heat-stress conditions.

Plants, as sessile organisms, encounter a range of biotic and abiotic stressors. Owing to the progression of global warming due to climate change, heat stress, characterised by above-suitable temperatures for plant growth, is considered one of the most serious abiotic stresses worldwide, leading to a reduction in crop yield (Zandalinas et al. 2021). Heat stress inhibits cellular and developmental homeostasis (Hasanuzzaman et al. 2013), exacerbating the reactive oxygen species (ROS) equilibrium imbalance in chloroplasts and mitochondria. This imbalance results in ROS accumulation, which causes cellular damage and genome instability similar to oxidative stress in plants (Baxter et al. 2014). Heat stress-induced protein misfolding and aggregation challenge the stability and function of protein complexes within plant cells, leading to the disruption of endoplasmic reticulum (ER) homeostasis, also known as ER stress (Kamauchi et al. 2005). Furthermore, prolonged exposure to high temperatures during reproductive development reduces fruit production and pollen viability in plants (Sato et al. 2006). Owing to the detrimental impact of heat stress on plant growth, plants have conserved systems of heat stress response (HSR), where heat shock factors (HSFs) and heat stress proteins (HSPs) play a central role in the maintenance of cellular homeostasis (Ding et al. 2020; Ohama et al. 2017). Consequently, researchers have developed transgenic plants expressing HSF or HSP to confer heat stress tolerance on several plant species. Overexpression of *Arabidopsis thaliana* (Arabidopsis) *HSFA2*, an HSF transcription factor crucial for inducing HSPs during HSR, confers enhanced heat-stress tolerance in plants (Nishizawa et al. 2006). Interestingly, the ectopic expression of *Brassica campestris* *HSFA1* (*BcHSFA1*) in *Nicotiana tabacum* (tobacco) resulted in improved growth under heat stress through the activation of HSPs, suggesting that the transcriptional network of the HSF family is conserved across various plant species (Zhu et al. 2018).

Enhancing heat-stress tolerance in crops through genetic engineering of HSR may seem appealing; however, this approach often requires specific transformation techniques for each plant species, resulting in lengthy development times. An alternative strategy to enhance resistance to environmental stresses in plants is chemical priming. This method involves activating the stress response using only functional chemical compounds, offering a promising way to maintain crop yield under stress conditions without genetic engineering (Sako et al. 2021).

Previously, we found that the non-proteogenic amino acid N-acetylglutamic acid (NAG) confers tolerance to oxidative stress in *Arabidopsis* and rice (Hirakawa et al. 2023). Gene expression analysis has shown that NAG activates oxidative stress-responsive genes, including antioxidant genes like *l-ascorbate peroxidase 1/2* (*APX1*/*2*), which are required for the maintenance of growth and seed production under heat stress in *Arabidopsis* (Suzuki et al. 2013). Because these findings suggest that NAG enhances heat-stress tolerance in plants, we examined whether NAG improves growth at high temperatures in plants.

The Arabidopsis plants used in this study were Col-0 accessions. Sterilised Arabidopsis seeds were incubated in distilled water at 4°C for 24 h. After incubation, seeds were sown in liquid medium containing 1/2 Murashige and Skoog (MS) medium and 1% sucrose (w/v), and then placed in an incubator set at 22°C with a 16 h light/8 h dark photoperiod. The chlorophyll content with heat stress in liquid culture was measured as previously described (Yamada et al. 2007; Yamaguchi et al. 2021). Seven-day-old Arabidopsis seedlings were treated with 0–0.1 mM NAG (NAG, Chemical Industry Co., Ltd., Tokyo, Japan) for 2 h, and then exposed to heat stress at 44°C for 1 h. After incubation at 22°C for 3 days, three seedlings were placed in 1 ml N,N′-dimethylformamide and kept at 4°C for 24 h. The absorbance of the extraction liquid was measured at 646.8 nm and 663.8 nm. Total chlorophyll content was calculated using the formula: Chl a+b (μM)=19.43 *A*_646.8_+8.05 *A*_663.8_. For soil planting experiment, Arabidopsis seeds were sown on Jiffy Seven (Sakata no Seeds, Ltd., Kanagawa, Japan), and placed in an incubator set at 22°C with a 16 h light/8 h dark photoperiod. The seedlings were watered with 0.5 mM NAG for 2 weeks. After 2 weeks, seedlings were incubated at 30°C with a 16 h light/8 h dark photoperiod for 1 week. Fresh weights of the aerial parts of the seedlings were measured.

For quantitative PCR (qPCR), total RNA was isolated from Arabidopsis seedlings using the RNeasy Plant Mini Kit (QIAGEN, Hilden, Germany) and extracted according to the manufacturer’s instructions. Genomic DNA was removed using an RNase-free DNase set (QIAGEN). Subsequently, cDNA was synthesised from 500 µg total RNA with Verso cDNA Synthesis Kit (Thermo Fisher Scientific, Waltham, MA, USA). For qPCR analysis, TB Green *Premix* *Ex Taq* II (Takara Bio Inc., Shiga, Japan) and a Light Cycler 480 (Roche, Basel, Switzerland) were used. The internal control was *ACTIN2*. The qPCR primers are listed in Supplementary Table S1. Chromatin immunoprecipitation (ChIP) was performed as previously described (Yamaguchi et al. 2014). Antibodies were added to the fraction of fragmented chromatins after preclearing, followed by overnight rotation at 4°C. Anti-H4ac (Merck Millipore:06-866) and anti-H3K4me3 (Abcam, ab8580) antibodies were used for immunoprecipitation. The qPCR analysis was performed using TB Green *Premix EX Taq* II and a Light Cycler 480. *TA3* served as a negative control. The qPCR primer sequences are listed in Supplementary Table S1.

The Nipponbare rice plant was used in this study. Sterilised rice seeds were incubated in liquid medium containing 1/2 MS medium in an incubator set at 30°C, with a 16 h light/8 h dark photoperiod. Three-day-old rice seedlings were treated with 0.5 mM NAG for 24 h and then exposed to heat stress at 44°C for 3 h. After incubation at 30°C for 3 days, the fresh weight of the shoots was measured.

To examine whether NAG enhances heat-stress tolerance, we examined the phenotype of Arabidopsis plants treated with NAG under heat stress conditions. Our experimental setup subjected the seedlings to heat stress (44°C, 1 h), resulting in reduced fresh weight and induced chlorosis in seedlings, with a reduction in chlorophyll content (Figure 1A–C). However, exogenous treatment with NAG rescued the reduction in fresh weight and chlorophyll content caused by heat stress in a dose-dependent manner (Figure 1B, C). Furthermore, we investigated whether NAG conferred heat-stress tolerance in plants during soil planting. In our soil-planting system, where plants were treated with prolonged, moderate heat stress (30°C, for 1 week), the fresh weight of NAG-treated seedlings was higher than that of untreated seedlings, but NAG was not observed to have any effect on the growth of seedlings under control conditions (Figure 1D, E, Supplementary Figure S1). These results suggest that exogenous treatment with NAG could alleviate heat stress in Arabidopsis.

**Figure 1:**
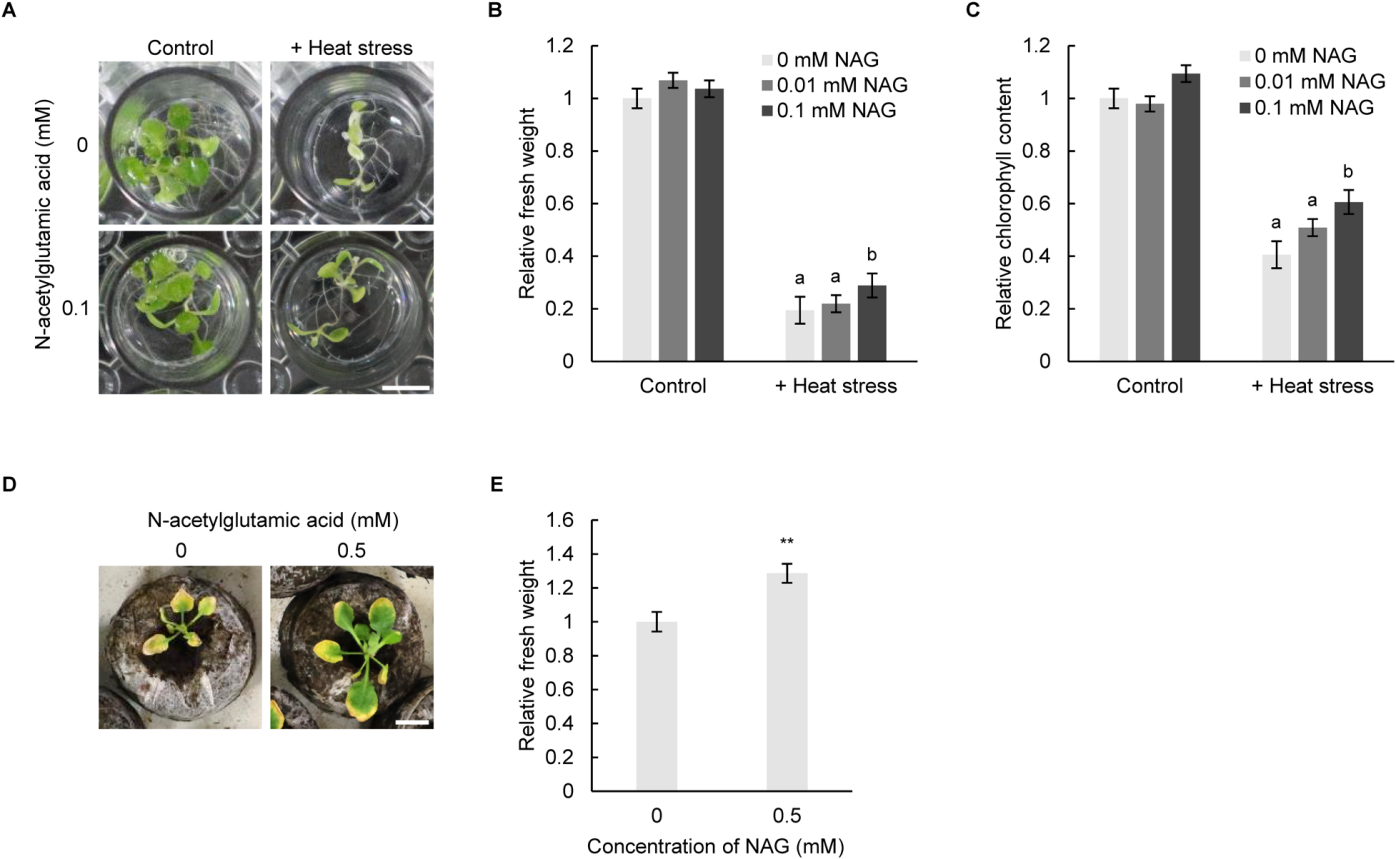
Figure 1. N-acetylglutamic acid alleviates heat stress in *Arabidopsis thaliana*. (A) Arabidopsis seedlings treated with 0.1 mM N-acetylglutamic acid (NAG) for 2 h, with or without heat stress (44°C, 1 h). Scale bar: 5 mm. (B) Fresh weight of seedlings treated with 0.01 mM and 0.1 mM NAG for 2 h, with or without heat stress. Error bars indicate standard error. *n*=9, *p*<0.05 (Tukey test). The letters indicate whether the difference is statistically significant. (C) Chlorophyll content of seedlings treated with 0.01 mM and 0.1 mM NAG for 2 h, with or without heat stress. Chlorophyll content (mM mg^−1^) was determined relative to the chlorophyll content of seedlings treated with 0 mM NAG and without heat stress. Error bars indicate standard error. *n*=9, *p*<0.05 (Tukey test). (D) Seedlings treated with 0.5 mM NAG upon prolonged heat stress (30°C, 1 week). Scale bar: 1 cm. (E) Fresh weight of seedlings treated with 0.5 mM NAG upon prolonged heat stress. The fresh weight of NAG-treated seedlings was compared to that of untreated seedlings. Error bars indicate standard error. *n*=30, ** *p*<0.01 (Student’s *t*-test).

In previous studies, we revealed that NAG increases the expression of oxidative stress-responsive genes through histone H4 acetylation (H4ac), an active histone modification that reduces ROS accumulation (Hirakawa et al. 2023). Therefore, we examined whether NAG treatment could also enhance the expression levels of heat stress-responsive genes. qPCR analysis showed that NAG treatment increased the expression of transcriptional factor *HSFA2* in non-heat stress conditions (Figure 2A). Because the expression levels of *HSFA2* were not activated in seedlings treated with NAG under heat stress, heat stress was not required for the induction of *HSFA2* with NAG treatment. The expression levels of *HSP21*, *HSP22*, *HSP17.6A* and *HSP18.2*, which are required for priming to heat stress downstream of *HSFA2*, did not increase in plants treated with NAG in either non-heat stress or heat stress conditions (Lämke et al. 2016; Olas et al. 2021; Yamaguchi et al. 2021) (Supplementary Figure S2). Next, to explore whether NAG alters histone modifications at the *HSFA2* locus, we conducted ChIP-qPCR using specific antibodies for H4ac and histone H3 lysine 4 trimethylation (H3K4me3), which are closely associated with gene-expression activation. ChIP-qPCR results indicated higher levels of histone H4ac but not H3K4me3 at the *HSFA2* locus in NAG-treated seedlings compared to those that did not undergo NAG treatment (Figure 2B). Thus, these results suggested that exogenous treatment with NAG specifically enhances *HSFA2* expression by enriching histone acetylation, conferring tolerance to heat stress.

**Figure 2:**
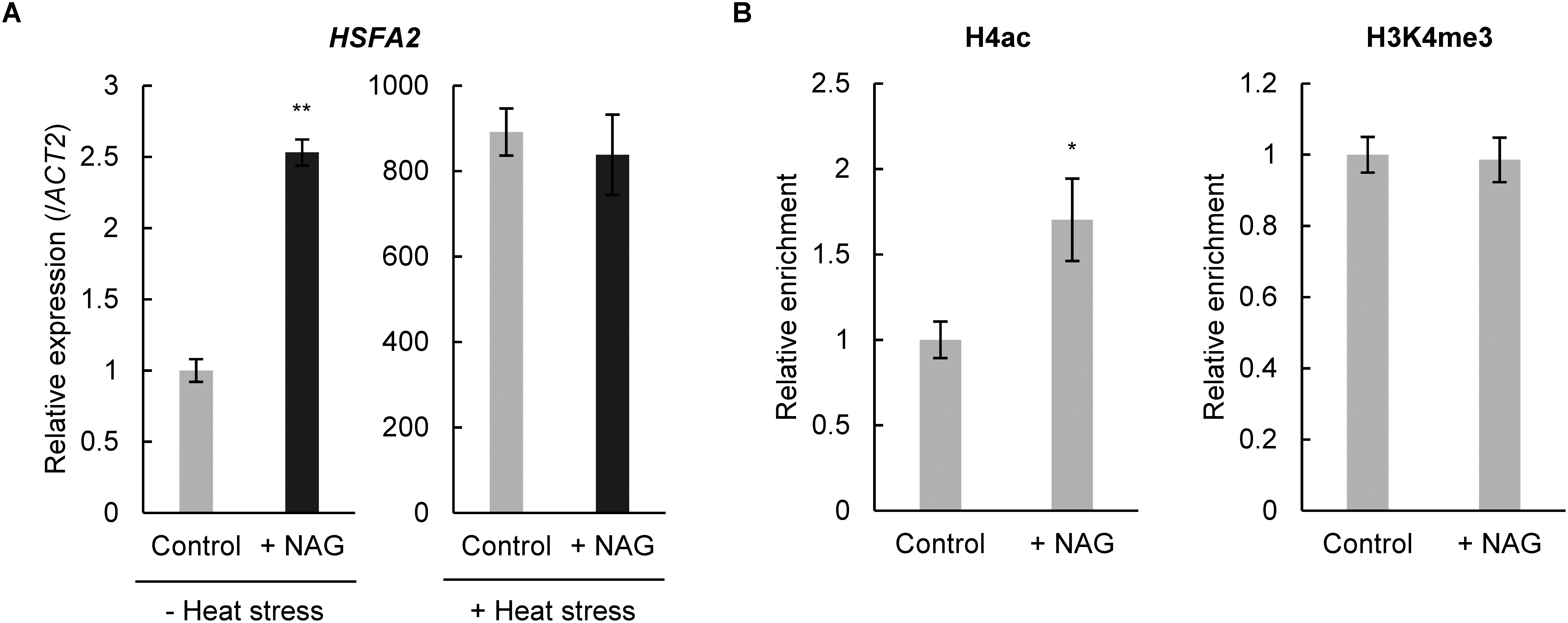
Figure 2. Expression levels and histone acetylation of *HSFA2* increase following treatment with N-acetylglutamic acid. (A) Expression levels of *HSFA2* in Arabidopsis seedlings treated with 0.1 mM N-acetylglutamic acid (NAG) for 2 h, with or without heat stress (44°C, 1 h). *n*=3, ** *p*<0.01 (Student’s *t*-test). (B) Histone H4 acetylation and H3K4me3 methylation levels at *HSFA2* of seedlings treated with NAG for 2 h. *n*=3, * *p*<0.05 (Student’s *t*-test).

To confirm the effectiveness of NAG in alleviating heat stress in both monocots and dicots, we examined the response of NAG-treated rice seedlings to heat stress. Our experiments showed that heat stress (44°C, 3 h) resulted in a reduction in the fresh weight of rice seedlings (Figure 3A, B). However, exogenous application of NAG suppressed the heat stress-induced reduction in the fresh weight of the seedlings (Figure 3B). This result suggests that NAG confers heat-stress tolerance in both dicot and monocot plants.

**Figure 3:**
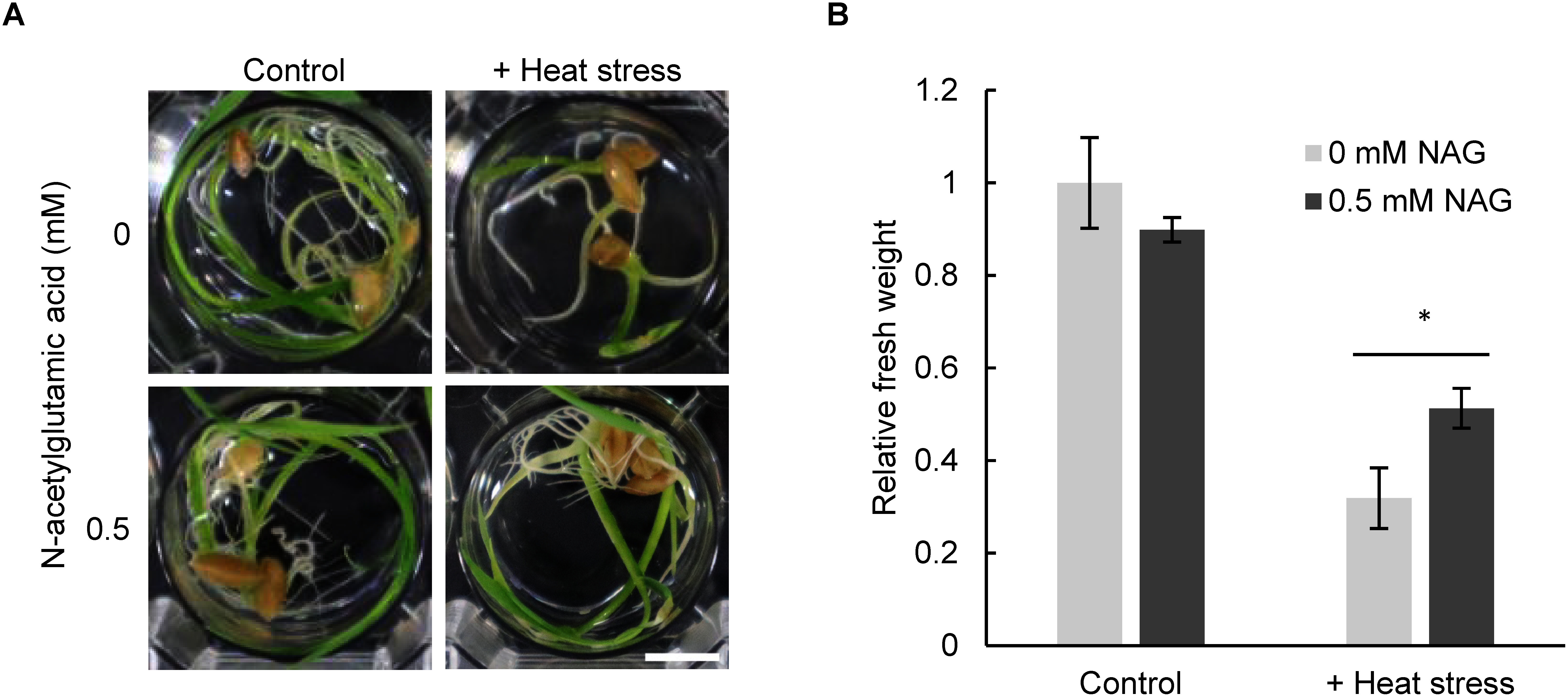
Figure 3. N-acetylglutamic acid confers tolerance to heat stress in *Oryza sativa*. (A) Rice seedlings treated with 0.5 mM N-acetylglutamic acid (NAG) for 24 h, with or without heat stress (44°C, 3 h). Scale bar: 10 cm. (B) Fresh weight of seedlings treated with 0.5 mM NAG for 24 h, with or without heat stress. *n*=9, * *p*<0.05 (Student’s *t*-test). Fresh weight was determined relative to the fresh weight of seedlings treated with 0 mM NAG and without heat stress.

In this study, we have demonstrated that exogenous treatment with NAG moderated heat stress in Arabidopsis and rice. Arabidopsis treated with NAG showed increased expression levels of *HSFA2* with the activation of H4ac (Figure 2B). In plants, histone acetylation is crucial for inducing global gene expression in response to environmental stresses, including heat stress (Hu et al. 2019). For example, heat stress in maize leads to global increases in histone acetylation, such as histone H3 lysine 9 (H3K9ac) and histone H4 lysine 5 (H4K5ac) (Wang et al. 2015). The histone acetyltransferase *GCN5* is essential for heat stress tolerance and the activation of heat stress-responsive genes, including *HSFA2* in Arabidopsis, under high temperatures (Hu et al. 2015). There are findings that metabolites having acetyl groups, such as acetic acid, are used as substrates for the acetylation of nucleotides in plants and bacteria (Kim et al. 2017; Taniguchi et al. 2018). Thus, NAG might activate heat stress-responsive genes with acetylation through the function of the acetyl group donor. In addition to histone acetylation, other histone and chromatin modifications influence the regulation of heat stress-responsive gene expression. The removal of the repressive histone modification histone H3 lysine 27 trimethylation (H3K27me3) by histone demethylases *JUMONJIs* (*JMJ30*, *JMJ32*, *REF6*, and *LEF6*) at *HSF* and *HSP* genes is necessary for their swift activation under heat stress, establishing a priming effect in Arabidopsis (Yamaguchi et al. 2021). Additionally, the Arabidopsis DNA binding factor *FGT1* collaborates with chromatin remodellers BRM, CHR11/17, and ISWI to reduce nucleosome occupancy at *HSF* and *HSP* genes, thereby facilitating their induction in response to heat stress (Brzezinka et al. 2016). These findings suggest that coordinated changes in epigenetic states and chromatin structures occur at *HSF* and *HSP* genes in plants under heat stress, allowing for an efficient heat-stress response.

Our qPCR analysis revealed that NAG increased the expression levels of *HSFA2* under non-heat stress conditions but not under heat stress conditions (Figure 2A). Previously, we found that Arabidopsis seedlings treated with NAG show enhanced expression of ROS-scavenging genes *alternative oxidase 1a* (*AOX1a*) and *APX1*/*2* (Hirakawa et al. 2023). Over-expression of *HSFA2* increases the expression level of ROS-scavenging genes including *APX2* without non-heat stress, conferring tolerance to heat stress (Banti et al. 2010). Expression levels of *HSP21*, *HSP22*, *HSP17.6A*, and *HSP18.2* which function in heat stress priming under *HSFA2*, were also not activated by NAG treatment in Arabidopsis (Supplementary Figure S2). These findings suggest that exogenous treatment with NAG might alleviate heat stress through *HSFA2*-mediated induction of ROS-scavenging genes before high-temperature conditions are encountered. Our soil planting experiments demonstrated that NAG significantly improved growth under prolonged and moderate heat-stress conditions (Figure 1D, E). Plants experience not only short-term heat stress but also long-term heat stress over extended periods. Arabidopsis mutants of *MIP3*, a subunit of the MAIGO2 tethering complex localised to the ER membrane, exhibit hypersensitivity to long-term heat stress and overactivation of ER stress-responsive genes (Isono et al. 2021). Similarly, the absence of *ELM1*, a regulator of mitochondrial fission with the dynamin complex, leads to an *AOX1a* expression and reduced tolerance to long-term heat stress in Arabidopsis (Tsukimoto et al. 2022). These results highlight the importance of maintaining organelle function and dynamics in the response to long-term heat stress and suggest that NAG might enhance tolerance to prolonged heat stress by regulating organelle homeostasis and gene expression in plants.

Heat stress often exacerbates the negative impact on plant growth when combined with other stresses, particularly high light stress, under field conditions (Medina et al. 2021). The combination of heat stress and high light stress significantly reduces photosynthetic rates more than each stress alone in Arabidopsis leaf (Balfagón et al. 2022). High light stress causes oxidative stress in plants by disrupting ROS equilibrium in organelles such as chloroplasts and mitochondria. Previously, we showed that exogenous treatment with NAG confers tolerance to oxidative stress as well as heat stress in plants (Hirakawa et al. 2023). Therefore, chemical priming with NAG offers the potential to improve crop yields under fluctuating environmental conditions by alleviating heat and oxidative stress.

## Abbreviations

*AOX1a*: *alternative oxidase 1a*
*APX1*/*2*: *l-ascorbate peroxidase 1/2*
*BcHSFA1*: *Brassica campestris HSFA1*
ChIP: chromatin immunoprecipitation
ER: endoplasmic reticulum
HSFs: heat shock factors
HSPs: heat stress proteins
HSR: heat stress response
H4ac: histone H4 acetylation
H3K4me3: histone H3 lysine 4 trimethylation
H3K9ac: histone H3 lysine 9 acetylation
H4K5ac: histone H4 lysine 5 acetylation
H3K27me3: histone H3 lysine 27 trimethylation
NAG: N-acetylglutamic acid
ROS: reactive oxygen species

## Description of Supplementary Files

Supplementary Table S1. List of primers used in this study.

Supplementary Figure S1. Exogenous treatment with N-acetylglutamic acid does not affect the growth of Arabidopsis in soil planting.

Supplementary Figure S2. Expression levels of *HSP21*, *HSP22*, *HSP17.6A* and *HSP18.2* in plants treated with NAG under both non-heat stress and heat stress.
