# Supplementary material for: Exogenous treatment with N-acetylglutamic acid confers tolerance to heat stress in plants
Source: Plant Biotechnol (Tokyo). 2024 Mar 25;41(1):71–6. doi: 10.5511/plantbiotechnology.23.1211a (PMC11500599; doi:10.5511/plantbiotechnology.23.1211a)
Supplement: Supplementary Data [file plantbiotechnology-41-1-23.1211a-s001.pdf]

Supplementary Table S1. List of primers used in this study.

Name	Sequence (5' → 3')	Experiment
ACTIN2_RT_F	GATCTCCAAGGCCGAGTATGAT	qPCR
ACTIN2_RT_R	CCCATTCATAAAACCCCAGC	qPCR
HSFA2_F	ATCAGCAAGGATCTGGGATG	qPCR
HSFA2_R	CCTCAGCTACAAGCACACCA	qPCR
HSP21_F	CAGGGCTCAAGACCAGAGAG	qPCR
HSP21_R	TCCCTTTTTGTTGTCCTTGC	qPCR
HSP22_R	GACTCGAGAGGGACACAAGC	qPCR
HSP22_R	TGCCCTTCTGCTGTTTCTTT	qPCR
HSP17.6A_F	ACCAGCTGACGTTATCGAGCA	qPCR
HSP17.6A_R	CACCACAAGCACGTTCTCGTT	qPCR
HSP18.2_F	TCCAAGCATTTTTGGAGGAC	qPCR
HSP18.2_R	TTTGTAAACGCTGCCACATC	qPCR
TA3_ChIP_F	CTGCGTGGAAGTCTGTCAAA	ChIP-qPCR
TA3_ChIP_R	CTATGCCACAGGGCAGTTTT	ChIP-qPCR
HSFA2_ChIP_F	TCCACGCCTCAATCTCTCAAC	ChIP-qPCR
HSFA2_ChIP_R	TGGGTTTTGGGTAAAGTTTGCG	ChIP-qPCR

A

B

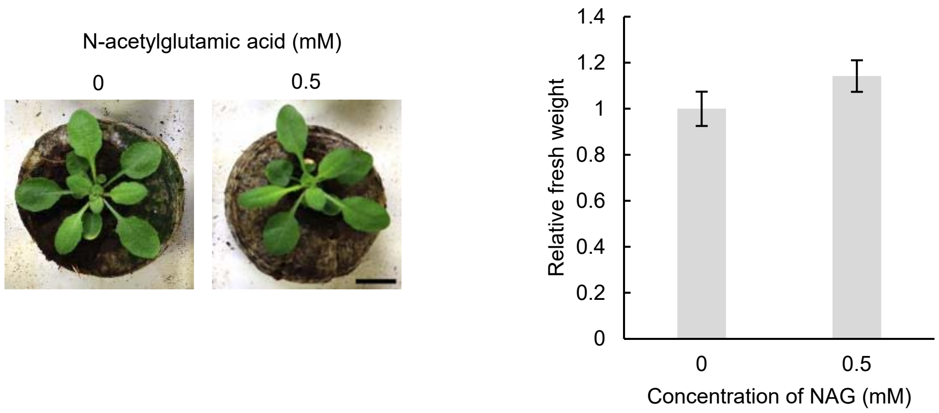

Supplementary Figure S1. Exogenous treatment with N-acetylglutamic acid does not affect the growth of Arabidopsis in soil planting.

(A) Arabidopsis seedlings treated with 0.5 mM N-acetylglutamic acid (NAG) in soil planting undernormal conditions (22 °C, 16 h light / 8 h dark). Scale bar: 1 cm. (B) Fresh weight of seedlings treated with 0.5 mM NAG in soil planting under normal conditions. The fresh weight of NAG-treated seedlings was compared to that of untreated seedlings. Error bars indicate standard error. n = 30.

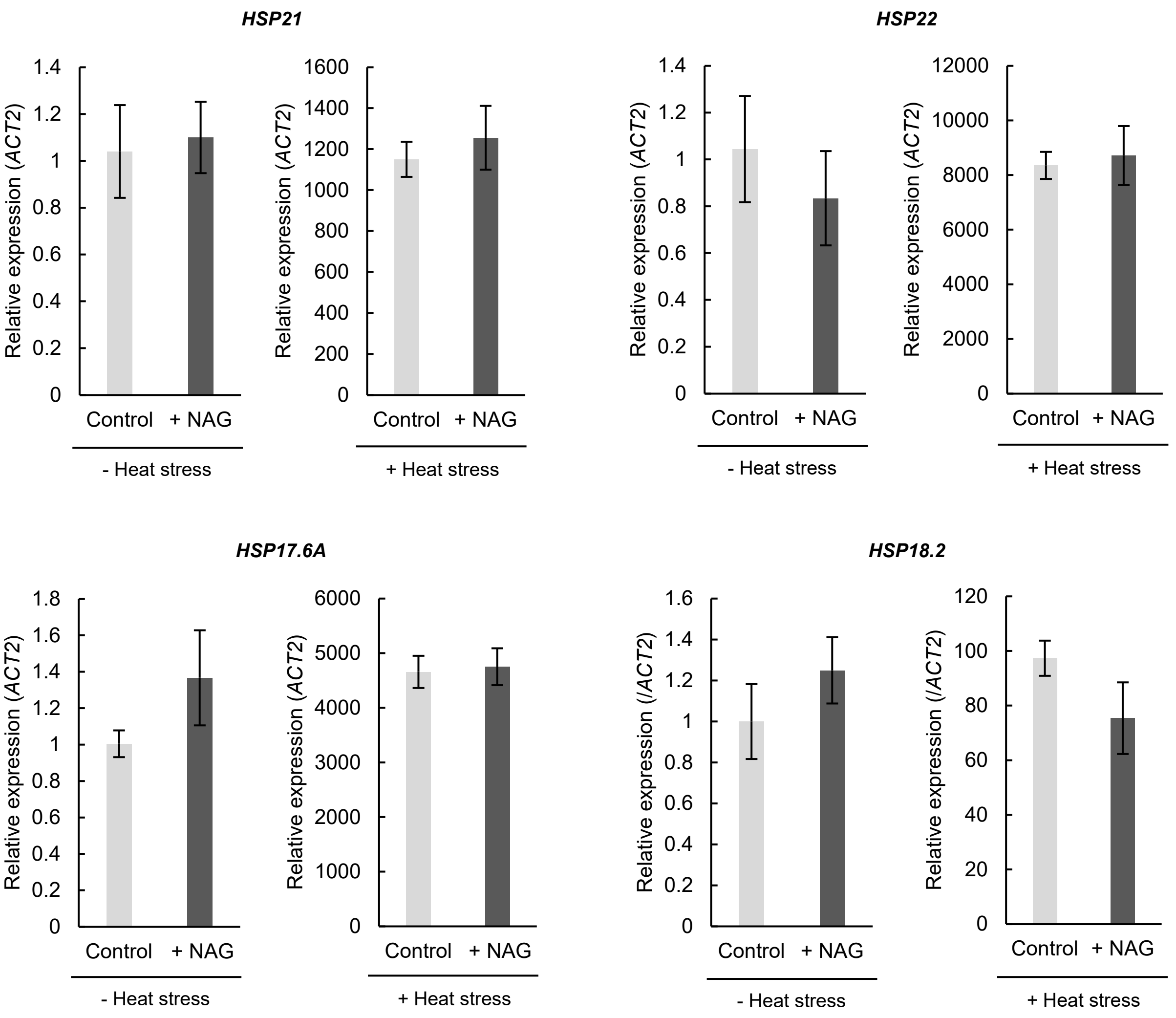

Supplementary Figure S2. Expression levels of *HSP21*, *HSP22*, *HSP17.6A* and *HSP18.2* in plants treated with NAG under both non-heat stress and heat stress.

Expression levels of *HSP21*, *HSP22*, *HSP17.6A* and *HSP18.2* in Arabidopsis seedlings treated with 0.1 mM N-acetylglutamic acid (NAG) for 2 h, with or without heat stress (44 °C, 1 h). Error bars indicate standard error. n = 3.
